# A telemonitoring programme in patients with heart failure in France: a cost-utility analysis

**DOI:** 10.1186/s12872-022-02878-1

**Published:** 2022-10-10

**Authors:** Mégane Caillon, Rémi Sabatier, Damien Legallois, Laurène Courouve, Valérie Donio, Florence Boudevin, Thibault de Chalus, Karine Hauchard, Annette Belin, Paul Milliez

**Affiliations:** 1 Amgen, Boulogne-Billancourt, France; 2 Service de Cardiologie et de Pathologie Vasculaire, CHU de Caen Normandie, Caen, France; 3 Université de Caen-Normandie, Caen, France; 4 APRIC (Association pour l’Amélioration de la Prise en charge de l’Insuffisance Cardiaque), Ouistreham, France; 5 Cemka, Bourg-la-Reine, France; 6 Normand’e-santé, Caen, France

**Keywords:** Telemonitoring, France, Heart failure, Cost-effectiveness

## Abstract

**Background:**

Certain telemedicine programmes for heart failure (HF) have been shown to reduce all-cause mortality and heart failure-related hospitalisations, but their cost-effectiveness remains controversial. The SCAD programme is a home-based interactive telemonitoring service for HF, which is one of the largest and longest-running telemonitoring programmes for HF in France. The objective of this cost-utility analysis was to evaluate the cost-effectiveness of the SCAD programme with respect to standard hospital-based care in patients with HF.

**Methods:**

A Markov model simulating hospitalisations and mortality in patients with HF was constructed to estimate outcomes and costs. The model included six distinct health states (three ‘not hospitalised’ states, two ‘hospitalisation for heart failure’ states, both depending on the number of previous hospitalisations, and one death state). The model lifetime in the base case was 10 years. Model inputs were based on published literature. Outputs (costs and QALYs) were compared between SCAD participants and standard care. Deterministic and probabilistic sensitivity analyses were performed to assess uncertainty in the input parameters of the model.

**Results:**

The number of quality-adjusted life years (QALYs) was 3.75 in the standard care setting and 4.41 in the SCAD setting. This corresponds to a gain in QALYs provided by the SCAD programme of 0.65 over the 10 years lifetime of the model. The estimated total cost was €30,932 in the standard care setting and €35,177 in the SCAD setting, with an incremental cost of €4245. The incremental cost-effectiveness ratio (ICER) for the SCAD programme over standard care was estimated at €4579/QALY. In the deterministic sensitivity analysis, the variables that had the most impact on the ICER were HF management costs. The likelihood of the SCAD programme being considered cost-effective was 90% at a willingness-to-pay threshold of €11,800.

**Conclusions:**

Enrolment of patients into the SCAD programme is highly cost-effective. Extension of the programme to other hospitals and more patients would have a limited budget impact but provide important clinical benefits. This finding should also be taken into account in new public health policies aimed at encouraging a shift from inpatient to ambulatory care.

**Supplementary Information:**

The online version contains supplementary material available at 10.1186/s12872-022-02878-1.

## Background

The management of patients with chronic heart failure (HF) is challenging due to the unpredictable occurrence of acute episodes of rapid onset, which can be life-threatening. In addition, patients with HF tend to be elderly and may have reduced mobility due to their disease, which can compromise timely and effective follow-up. For these reasons, HF is a promising candidate for the implementation of telemedicine programmes that allow patients to be managed at home. Such programmes have now been implemented in many different countries for over 20 years. Even though the nature of these programmes varies considerably, recent systematic reviews have generally concluded that intense programmes, notably those with dedicated nurse follow-up, can reduce all-cause mortality and heart failure-related hospitalisations [1–5].

Although providing important clinical benefits, effective telemedicine programmes carry a cost, and it is thus important to evaluate whether they are cost-effective for the management of HF. A number of economic studies have evaluated clinical outcomes and costs of these programmes [6–17], and provided somewhat inconsistent results. A recent systematic review has nonetheless concluded that telemonitoring programmes in HF are cost-effective, with their greatest impact and cost savings through reduced rehospitalisations [5].

Currently, health authorities in many countries are considering implementing and reimbursing telemedicine programmes for different chronic diseases with the goal of reducing the demand on hospital services and generating cost savings. In addition, the recent COVID-19 pandemic has illustrated the attractiveness of telemedicine interventions for managing patients with chronic diseases in their homes [18]. To this end, it is important for health authorities to dispose of national economic evaluations for individual telemonitoring programmes in the disease area of interest. The French health authorities are currently assessing the value of introducing a fully reimbursed telemonitoring programme at the national level for routine management of patients with HF. One of the candidate programmes for extension nationwide is the SCAD (*Suivi Clinique A Domicile*) programme, a home-based interactive telemonitoring service for HF established in the French region of Normandy in 2007 [19]. The programme is offered to all patients hospitalised for an acute exacerbation of HF, who are entered into the programme prior to discharge. Between 2007 and 2016, around 1000 patients in Normandy have been enrolled in the programme. The benefits of the SCAD programme in terms of reduced hospitalisations and mortality have been demonstrated both in a randomised clinical trial [20] and in a naturalistic setting [19]. In an analysis of the French national health insurance database (*Système national des données de santé*; SNDS), the rate of unplanned hospitalisations for a cardiovascular diagnosis was halved in SCAD participants compared to non-participants, the rate of unplanned hospitalisations for HF divided by three, and mortality reduced by around twenty percent [19]. The objective of this cost-utility analysis was to evaluate the cost-effectiveness of the SCAD home telemonitoring programme with respect to standard hospital-based care in patients with HF in France.

## Methods

### Study design

This was a cost-effectiveness analysis comparing the use of the SCAD programme in patients with HF compared with standard care without telemonitoring in the French setting. The cost data from this study have been presented at the European Society for Cardiology Congress in August 2020 [21].

Outcomes and costs were generated in a Markov model simulating hospitalisations and mortality in patients with HF. The Markov model was developed in Microsoft Excel (release 2010, Microsoft Inc, USA). The analysis was performed from a collective perspective, taking into account direct medical and non-medical costs borne by the national health insurance (NHI), by complementary private health insurance and by the patient. The analysis complied with the recommendations of the French Health Authorities for economic evaluations of innovative health technologies [22]. A discount rate of 2.5% was applied, as recommended in these guidelines [22].

### SCAD programme

The SCAD programme is open to patients recently hospitalised for an acute exacerbation of heart failure in one of the participating hospital centres. Patients are enrolled into the programme mainly at the time of discharge from the hospital and remain in the programme for a period of three months, which can be renewed for a further three months if the patient and cardiologist so desire. Each patient is provided with a dedicated programme installed on a tactile pad for entering data and an internet link to the coordinating centre at the hospital. Over 6 days a week (Sunday excluded), the patient enters information on their clinical state (weight, blood pressure, heart rate and occurrence of cardiac symptoms), lifestyle factors (diet and physical activity), their psychological state (evaluation of fatigue and morale on a 10-point visual analogue score) and treatment compliance (assessed with an open question). The healthcare team can access the data entered by the patient through a secure internet portal during office hours. Personalised feedback is provided by a dedicated trained HF nurse from the hospital cardiology department through a telephone call or a text message. The data entered by the patient are also analysed in real-time by a risk algorithm in order to identify any risk of acute decompensation, and automatically generate an alert, if necessary. This enables the HF nurse to contact the patient, the general practitioner, the cardiologist or the emergency services whenever necessary in order to organise a consultation or a visit to the hospital. The computer interface also provides access for the patient to information and advice on treatment and on leading a healthy lifestyle. In addition, a secure chat tool is available for the patient throughout the duration of the programme which provides them with a direct contact with the care team and access to an information bank about heart failure. A full description of the organisation of the SCAD programme has recently been published [19].

### Description of the model

**Fig. 1:**
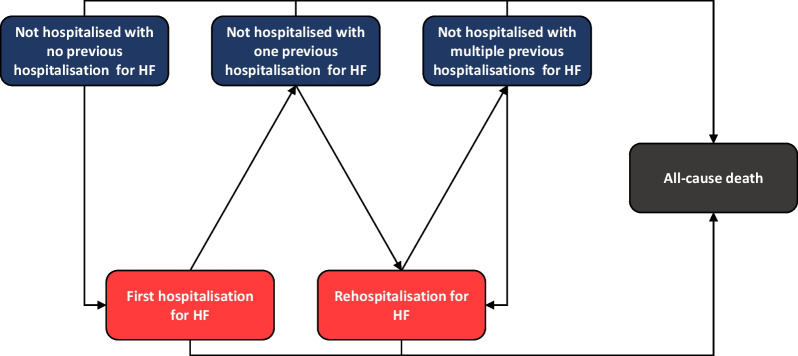
Structure of the Markov model

A Markov model was developed to simulate six distinct health states, classified as ‘not hospitalised’ (three possible states), ‘hospitalisation for heart failure’ (two possible states), both depending on the number of previous hospitalisations, and death (one state). The structure of the model is illustrated in Fig. 1. Patients entered the model in one of three ‘not hospitalised’ health states, depending on the number of previous hospitalisations for HF, namely no previous hospitalisation for HF in the previous year, a single previous hospitalisation for HF, or multiple previous hospitalisations. The distribution of patients across these three health states was determined from the number of hospitalisations documented in the SNDS with HF as a primary diagnosis (identifying the reason for hospitalisation) in a population of patients with chronic HF enrolled into the SCAD telemonitoring programme in France between 2009 and 2016 [19]. Hospitalisations for HF were identified using the diagnostic algorithm developed by Tuppin et al. [23]. The characteristics of the patients in this population, overall and by age, LVEF category and NYHA class, are presented in Additional file 1.

In the next cycle, patients can either remain in the same ‘not hospitalised’ health state, transit to a hospitalisation state or die. Two hospitalisation states were considered, either a first hospitalisation for HF, or rehospitalisation for HF. From a hospitalisation health state, patients could either revert to the corresponding ‘not hospitalised’ state or die. Patients could not remain in a hospitalisation health state for more than one cycle and were obliged to transit at the end of the cycle. Death was considered the end state for the model. The cycle length was one month. This was based on the anticipated average duration of hospitalisation for heart failure [24], as patients cannot stay hospitalised for more than one cycle. The time horizon of the analysis was ten years in order to capture the effects and direct costs of the telemonitoring programme. No intercurrent events, such as the occurrence of adverse events, were modelled, in the absence of information on how these might influence transition probabilities or outcomes.

### Model population

The analysis population modelled corresponded to a hypothetical cohort population of 10,000 adult patients with heart failure. This population size was chosen to match the incidence of first hospitalisation for heart failure derived from the SNDS [19].

### Transition probabilities

Transition probabilities between each health state were based on published literature. For the initial hospitalisation and mortality, these were estimated from data collected in the ODIN study [25]. This was a large, prospective, multicentre French cohort of 3237 patients recruited by 61 French centres between 2007 and 2010 and followed up until 2013 [25]. For rehospitalisation, the transition probabilities were based on a more recent analysis of data from the SNDS [26]. All-cause mortality rates for the French general population were obtained from the French national statistics office [27]. All transitions used in the model were stratified by age (< 70 years and ≥ 70 years) and by HF severity (New York Heart Association grade) and the transition probabilities in each stratum were adjusted by the relative risk of hospitalisation and death observed in the ODIN study. Patients remained in the same age and severity class throughout the lifetime of the model. All transition probabilities are listed in Table 1.

**Table 1:** Model inputs

N°	Variable	Category	Value	Source
*Model population*				
1	Age group	< 70 years	56.0%	SCAD cohort [19]
		≥ 70 years	44.0%	SCAD cohort [19]
2	Severity	NYHA I/II	61.0%^a^	SCAD cohort [19]
		NYHA III/IV	39.0%	SCAD cohort [19]
3	Extent of use of programme	Intermediate	50.4%	SCAD cohort [19]
		High	49.6%	SCAD cohort [19]
4	Number of hospitalisations for HF	0 in previous 12 months	52.1%	SCAD cohort [19]
		1 in previous 12 months	36.0%	SCAD cohort [19]
		≥ 2 in previous 12 months	11.9%	SCAD cohort [19]
*Transition probabilities*				
5	Not hospitalised with no previous hospitalisation for HF to first hospitalisation for HF		0.006	ODIN study [25]
6	Not hospitalised with one previous hospitalisation for HF to rehospitalisation for HF		0.017	AMELI study [28]
7	Not hospitalised with ≥ 2 previous hospitalisations for HF to rehospitalisation for HF		0.017	AMELI study [28]
8	Not hospitalised with no previous hospitalisation for HF to death		0.0066	ODIN study [25]
9	Not hospitalised with one previous hospitalisation for HF to death		0.0085	ODIN study [25]
10	Not hospitalised with ≥ 2 previous hospitalisations for HF to death		0.0181	ODIN study [25]
11	First hospitalisation for HF to death (assumption)		0.0085	Equivalent to n° 9
12	Rehospitalisation for HF to death (assumption)		0.0181	Equivalent to n° 10
*Adjustment factors* (*relative risk*)				
13	Age < 70 years and NYHA I/II: risk of hospitalisation for HF		0.79	ODIN study [25]
	Age < 70 years and NYHA III/IV: risk of hospitalisation for HF		1.43	ODIN study [25]
	Age ≥ 70 years and NYHA I/II: risk of hospitalisation for HF		0.74	ODIN study [25]
	Age ≥ 70 years and NYHA III/IV: risk of hospitalisation for HF		1.32	ODIN study [25]
14	Age < 70 years and NYHA I/II: risk of death		0.76	ODIN study [25]
	Age < 70 years and NYHA III/IV: risk of death		1.48	ODIN study [25]
	Age ≥ 70 years and NYHA I/II: risk of death		0.71	ODIN study [25]
	Age ≥ 70 years and NYHA III/IV: risk of death		1.36	ODIN study [25]
*Efficacy of intervention* (*relative risk*)				
15	Risk of hospitalisation for HF		0.500	SCAD cohort [19]
	Risk of death		0.535	SCAD cohort [19]

*HF* heart failure; *NYHA* New York Heart Association class

^a^Data on NYHA class were unavailable for 9.1% of patients in the study

### Interventions compared

The Markov model compared costs and outcomes between patients participating in the SCAD programme and non-participants (standard care setting). The relative risk of events (hospitalisation or death) for participants compared to non-participants was applied to the transition probabilities in the model. Relative risks were estimated from outcomes reported in a recent study in which 659 SCAD participants were linked to the SNDS database [19] (Table 1). In this study, three groups of patients who differed according to the extent of use of the SCAD programme (low, intermediate and high users) were defined by tercile and outcomes in each group analysed over the follow-up period [19]. However, there was no control group of patients with HF not participating in the programme.

The effectiveness of the telemonitoring programme varies with the extent of use, being greatest in the high users. Since there was no control group of patients with HF not participating in the programme, low users (including no users) were considered to represent non-participants and attributed a relative risk of effectiveness of 1. The efficacy of the SCAD programme was expressed as the relative risk of HF-related hospitalisation or of death for intermediate/high users versus low users of SCAD. The relative risks were estimated from Kaplan–Meier analysis of event rates. The survival curves are provided in Additional file 2. It was assumed that effectiveness was constant for the first 60 months of the model, as shown from a proportional hazard analysis of the Kaplan–Meier curves [19]. Over the next five years the SCAD programme was considered to be no longer effective, since no data is available beyond 60 months. These relative risks were then applied to the transition probabilities to generate use-level specific probabilities for intermediate/high users.

### Utilities

Utilities for the model were taken from a cost-utility analysis of data from the Systolic HF Treatment with the If Inhibitor Ivabradine Trial (SHIFT) [29, 30]. This analysis reported quality of life (QoL) data using the EuroQoL (EQ-5D) questionnaire, which was administered to 5313 patients. Using the data, a multilevel regression analysis was performed in order to estimate the variation in EQ-5D as a function of age, gender, NYHA class and hospitalisation status [31]. The regression coefficients were used to weight the baseline utility values of the four different patient subgroups (age < 70 years or ≥ 70 years, NYHA class I/II or III/IV) and to determine the disutility value for hospitalisation to be used in the Markov model. These derived utility values are presented in Table 2.

**Table 2:** Utility data considered in the model

Description	Baseline utility
< 70 years & NYHA I/II	0.788
< 70 years & NYHA III/IV	0.669
≥ 70 years & NYHA I/II	0.749
≥ 70 years & NYHA III/IV	0.603
Hospitalisation	− 0.212

*NYHA* New York Heart Association class

### Costs

Cost inputs were derived from the SCAD cohort. Individual cost items were retrieved from the SNDS for the 528 patients included from 2010 onwards and who survived for at least twelve months [19] [21]. The reason for excluding patients enrolled prior to 2010 was to ensure collection of exhaustive data on healthcare resource utilisation, since complete information has only been available in the database since 2009. Patients with less than one year’s follow-up were excluded in order to enable annual costs to be determined accurately. The procedure for cost estimation complied with the guidelines of the French Health Authorities [22]. Hospitalisation costs were estimated using the National Reference System for Hospital Costs (2016 tariffs, applicable at the time the study was initiated), which provides consolidated unit costs for individual stays according to the reason for hospitalisation, defined by ICD-10 diagnostic groups. All costs were adjusted for inflation and are presented as 2021 Euros.

Unit costs for all healthcare resource expenditure items are provided in Additional file 3. Consolidated costs associated with all states in the Markov model are presented in Table 3. Management costs for the three ‘not hospitalised’ HF states (0, 1 or ≥ 2 previous hospitalisations) were estimated from the median community and outpatient costs accrued over the 12 months before enrolment into the SCAD programme for the patients in the ‘standard care’ setting and over the 12 months following enrolment for SCAD participants. Only those management costs that differed significantly between the two settings were integrated into the model. The cost of hospitalisation was determined individually according to the level of adherence to the SCAD programme (low, intermediate, high). The overall unit hospitalisation cost for patients in the SCAD setting was calculated from costs in the intermediate and high user groups, weighted by the distribution of patients across the two groups. Unit hospitalisation cost for patients in the standard care setting corresponded to that estimated for low users. The end-of-life cost was the cost of palliative care in the last three months before death. The cost of the SCAD programme was based on the tariffs billed by the hospital, which are specified by the national programme for the evaluation of telemedicine of the French Health Ministry (ETAPES programme). For HF, this corresponds to a 6 months renewable care package costed at €470 for 6 months. This tariff covers €300 for the supplier, €110 for the cardiologist and €60 for therapeutic education. In the model, a monthly cost of €78,33 was applied over a 6 months period.

**Table 3:** Costs considered in the model

Resource	Time period considered	Unit cost (€2021)
Cost of SCAD programme	Monthly for 6 months	€78.33
Management cost for non-hospitalised patients: standard care	Monthly	€197.81
Management cost for non-hospitalised patients: SCAD	Monthly	€268.52
HF hospitalisation cost: standard care (low SCAD use)	Individual stay	€7138.29
HF hospitalisation cost: SCAD users (weighted^1^)	Individual stay	€5742.21
HF hospitalisation cost: intermediate SCAD user	Individual stay	€5877.70
HF hospitalisation cost: high SCAD user	Individual stay	€5604.33
Palliative care cost	Monthly for 3 months before death	€20,847.11

*HF* heart failure; *SCAD* *Suivi Clinique A Domicile*

^1^Weighted to take into account the patient mix between high and intermediate users

### Model outputs

Outcomes were modelled as life-years (LYs), quality-associated life years (QALYs) and total cost for each management strategy (SCAD and standard care). Incremental differences in costs and QALYs between the SCAD programme and standard care were calculated and the incremental cost-effectiveness ratio (ICER) derived by dividing the incremental cost by the incremental effectiveness. ICERs were calculated for both QALYs and LYs.

### Sensitivity analysis

Deterministic and probabilistic sensitivity analyses were performed to assess uncertainty in the input parameters of the model. In the deterministic sensitivity analysis (DSA), key model inputs were varied within their standard errors or 95% confidence intervals. If empirical data to inform these precision estimates were unavailable, an arbitrary range of ± 20% was applied. The variables used in the deterministic sensitivity analysis (DSA) and the range of values applied are listed in Table 4.

**Table 4:** Deterministic sensitivity analysis: range of values tested

Parameter	Base case	Lower bound	Upper bound
Duration of participation in the SCAD programme (months)	6	− 20%	+ 20%
TP No previous hospitalisation for HF to death	0.006	− 20%	+ 20%
TP One previous hospitalisation for HF to death	0.008	− 20%	+ 20%
TP Two previous hospitalisations for HF to death	0.018	− 20%	+ 20%
TP First hospitalisation for HF to death	0.008	− 20%	+ 20%
TP Rehospitalisation for HF to death	0.018	− 20%	+ 20%
TP No previous hospitalisation for HF to first hospitalisation for HF	0.006	− 20%	+ 20%
TP One previous hospitalisation for HF to rehospitalisation for HF	0.017	− 20%	+ 20%
TP Two previous hospitalisations for HF to rehospitalisation for HF	0.017	− 20%	+ 20%
TP risk adjustment HF hospitalisation < 70 years NYHA I/II	0.794	− 20%	+ 20%
TP risk adjustment HF hospitalisation < 70 years NYHA III/IV	1.428	− 20%	+ 20%
TP risk adjustment HF hospitalisation ≥ 70 years NYHA I/II	0.745	− 20%	+ 20%
TP risk adjustment HF hospitalisation ≥ 70 years NYHA III/IV	1.324	− 20%	+ 20%
TP risk adjustment death < 70 years NYHA I/II	0.756	− 20%	+ 20%
TP risk adjustment death < 70 years NYHA III/IV	1.482	− 20%	+ 20%
TP risk adjustment death ≥ 70 years NYHA I/II	0.706	− 20%	+ 20%
TP risk adjustment death ≥ 70 years NYHA III/IV	1.360	− 20%	+ 20%
Relative risk of hospitalisation for HF due to SCAD	0.5	− 20%	+ 20%
Persistence of effectiveness of SCAD programme (months)	60	90	120
Relative risk of death due to SCAD	0.535	− 20%	+ 20%
Utility ≥ 70 years NYHA I/II	0.749	− 20%	+ 20%
Utility under70 NYHA III/IV	0.669	− 20%	+ 20%
Utility under70 NYHA I/II	0.788	− 20%	+ 20%
Utility ≥ 70 years NYHA III/IV	0.603	− 20%	+ 20%
HF hospitalisation cost with SCAD	5742	− 20%	+ 20%
HH hospitalisation cost without SCAD	7138	− 20%	+ 20%
Management cost without SCAD (monthly)	197.8	− 20%	+ 20%
Management cost with SCAD (monthly)	268.5	− 20%	+ 20%
Palliative care cost	20,847	− 20%	+ 20%
Discount rate	2.5%	1.5%	4%

*NYHA* New York Heart Association class; *SCAD* *Suivi Clinique A Domicile*; *TP* transition probability

In the probabilistic sensitivity analysis (PSA), Monte Carlo simulations were performed allowing the values for the model inputs to vary according to their sampling distributions (5000 iterations). A log-normal distribution was applied for clinical data, a beta distribution for utilities and a gamma distribution for costs. The results of the analysis are expressed as the cost-effectiveness acceptability curve for the net benefit.

### Scenario analysis

Five scenario analyses were also performed. The first two evaluated cost-effectiveness in different patient subgroups and the remaining analyses evaluated changes in the parameters defining the structure of the model. In the first analysis, the model was reiterated in three subgroups of patients categorised by their left ventricular ejection fraction (LVEF). These were preserved ejection fraction (pEF: > 50%), mid-range ejection fraction (mrEF: 40–49%) and reduced ejection fraction (rEF: < 40%). Transition probabilities for initial hospitalisation and mortality according to LVEF class were based on data from the ODIN cohort [25]. Since rehospitalisation rates according to the type of ejection fraction are not available in the study of the SNDS [26], the same transition probabilities were used for all three LVEF categories as in the base-case analysis. Transition probabilities and relative risks of clinical outcomes used in this scenario analysis are provided in Additional file 4 and Additional file 5.

The second scenario analysis focused on subgroups of patients with mild-to-moderate (NYHA class I or II) and severe (NYHA class III or IV) heart failure. Transition probabilities and relative risks of clinical outcomes used in this scenario analysis are provided in Table 1.

In the third scenario analysis, the time horizon of the model was set at either five years or for the lifetime of the patient (compared to ten years in the base-case analysis). The five-year horizon was chosen as this is the length of time for which the SCAD programme has been shown to be effective at reducing hospitalisation and mortality [19]. ‘Lifetime’ was considered to be the time period between entry into the model and transition to the death state for each patient.

In the fourth scenario analysis, all patients entered the model in the ‘not hospitalised after one previous hospitalisation’ state. The justification for this is that the SCAD programme is only proposed to patients at the time of hospital discharge. In the base case, patients who enter the model in the ‘not hospitalised with no previous hospitalisation’ state and who are not hospitalised during the lifetime of the model will never be proposed the SCAD programme. The scenario analysis evaluates a scenario in which all patients are proposed the programme.

The final scenario analysis evaluated the situation in which participation in the SCAD programme was continued over the entire 10 years period over which costs and utilities were determined.

## Results

### Utility outcomes

Over the course of the model, the total number of hospitalisations was 0.749 in the standard care setting and 0.612 in the SCAD setting (Table 5). The number of life years was 5.11 and 6.03 respectively. The number of quality-adjusted life years (QALYs) was 3.75 in the standard care setting and 4.41 in the SCAD setting. This corresponds to a gain in QALYs provided by the SCAD programme of 0.65 over the 10 years lifetime of the model (Table 5).

**Table 5:** Utility and cost outcomes

	SCAD	Standard care	Incremental
Total number of hospitalisations for HF per patient	0.612	0.749	− 0.137
First hospitalisations for HF	0.156	0.186	− 0.030
Rehospitalisations for HF	0.456	0.563	− 0.108
Life years	6.03	5.11	0.93
QALYs	4.41	3.75	0.65
Total Costs	€35,177	€30,932	€4,245
SCAD costs	€461	–	€461
HF-specific management costs	€19,251	€11,954	€7297
HF hospitalisation costs	€3120	€4875	€1755
Palliative care costs	€12,345	€14,103	€1758
ICER (€/LY)			€4579
ICER (€/QALY)			€6491

*HF* heart failure; *ICER* incremental cost-effectiveness ratio; *LY* life year; *QALY* quality-adjusted life year; *SCAD* *Suivi Clinique A Domicile*

### Cost outcomes

The estimated total cost was €30,932 in the standard care setting and €35,177 in the SCAD setting, with an incremental cost of €4245 (Table 5). The largest component of costs were the HF-specific management costs. The higher incremental cost in the SCAD setting was principally due to higher total management costs as the patients survived longer in this setting. The cost of the SCAD programme itself contributed < 2% of the total cost and around 10% of the incremental cost (Table 5).

### Cost-utility

The ICER was €4579 in terms of incremental cost per life year gained and €6491 in terms of incremental cost per quality-adjusted life year gained (Table 5).

### Sensitivity analysis

**Fig. 2:**
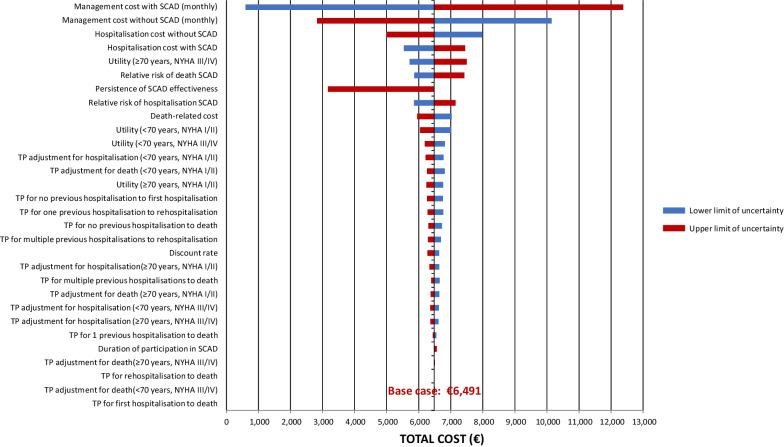
Deterministic sensitivity analysis. Blue: lower limit of uncertainty; red: upper limit of uncertainty. All hospitalisation items in this analysis are restricted to hospitalisations for heart failure, as specified in Table 4

A deterministic sensitivity analysis was performed to identify variables that influenced the ICER of the SCAD programme compared to standard care. The results are presented in the form of a tornado plot in Fig. 2. The variables that had the most impact on the ICER were HF management costs, both in the standard care setting and the SCAD setting. Other variables whose precision influenced the ICER were hospitalisation costs, the utility value for patients aged ≥ 70 years in NYHA class III/IV and the persistence of the effectiveness of the SCAD programme. Varying the values of the other variables in either direction did not change the estimated ICER by more than €500/QALY.

**Fig. 3:**
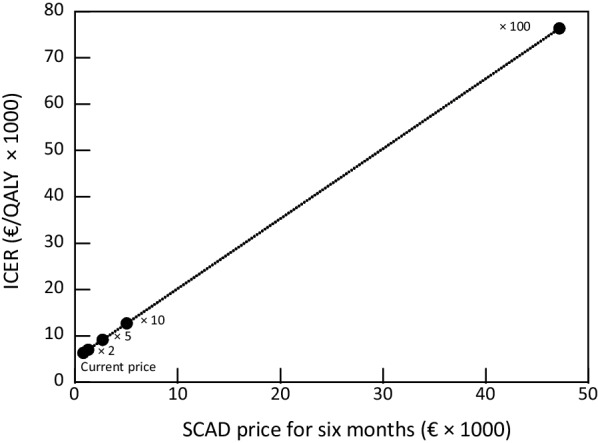
Effect on the ICER of varying SCAD cost

The effect of increasing the cost of the SCAD programme on the estimated ICER is illustrated in Fig. 3. A one-100-fold increase in cost would lead to an increase in the ICER to €76,280.

**Fig. 4:**
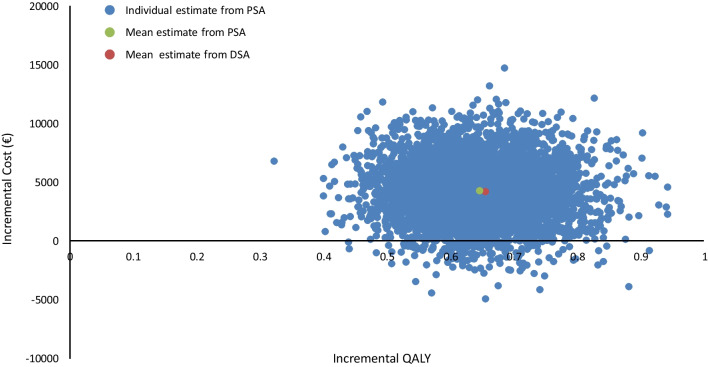
Probabilistic sensitivity analysis. Each blue point represents the incremental cost (€) and utility (QALY) of the SCAD programme from an individual Monte Carlo simulation. The grey point represents the mean incremental cost and utility derived from all Monte Carlo simulations and the orange point the incremental cost and utility derived from the deterministic base case analysis.

**Fig. 5:**
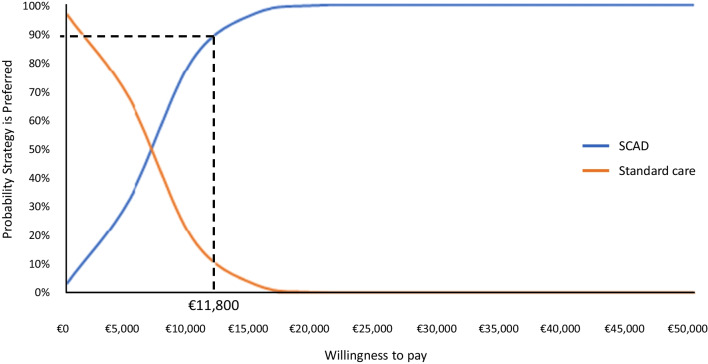
Willingness to pay thresholds. Blue curve: SCAD programme; orange curve: standard care.

In the probabilistic sensitivity analysis, the mean incremental cost of the SCAD programme generated from the Monte Carlo simulations was €4314 and the mean incremental utility gained was 0.64 QALYs, corresponding to an ICER for the SCAD programme of €6689 €/QALY with a standard deviation of €3883. These values are very close to those observed in the base-case analysis. A scatter diagram of the outputs from the individual Monte Carlo simulations is presented in Fig. 4. The distribution of the outputs was symmetrical and centred on the mean values. It should be noted that in 3% of simulations, the SCAD strategy was dominant (less expensive and more effective than standard of care). A cost-effectiveness acceptability curve was plotted based on the PSA, which showed that the likelihood of the SCAD programme being considered cost-effective was 90% at a willingness-to-pay threshold of €11,800 (Fig. 5).

### Scenario analyses

The outcomes of the scenario analyses based on patient subgroups are presented in Table 6. In the analysis of patients with different LVEF, the ICERs in the three scenario analyses ranged from €5843/QALY in patients with preserved ejection fraction to €6625/QALY in those with mid-range ejection fraction. Variation in both cost and utility outcome contributed to this difference.

**Table 6:** Scenario analyses in patient subgroups

	Life years	QALYs	Total costs (€)	ICER (€/LY)	ICER (€/QALY)
*Base case*					
SCAD	6.03	4.41	35,177		
SC	5.11	3.75	30,932	4579	6491
Difference	0.93	0.65	4245		
*Preserved ejection fraction*					
SCAD	5.88	4.24	34,707		
SC	4.87	3.54	30,628	4045	5843
Difference	1.01	0.70	4079		
*Mid-range ejection fraction*					
SCAD	6.19	4.54	34,107		
SC	5.29	3.90	29,821	4741	6625
Difference	0.90	0.65	4287		
*Reduced ejection fraction*					
SCAD	6.00	4.32	35,781		
SC	5.03	3.65	31,448	4474	6431
Difference	0.97	0.67	4332		
*NYHA Class I/II*					
SCAD	6.66	5.16	34,694		
SC	5.87	4.55	30,144	5779	7500
Difference	0.79	0.61	4550		
*NYHA Class III/IV*					
SCAD	5.06	3.23	35,933	3289	5176
SC	3.91	2.51	32,164		
Difference	1.15	0.73	3769		

*ICER* incremental cost-effectiveness ratio; *LY* life-years; *NYHA* New York Hospital Association; *QALY* quality-adjusted life year; *SC* standard care; *SCAD* *Suivi Clinique A Domicile*

In the scenario analysis evaluating different severity groups (Table 6), the QALYs gained were higher in the NYHA I/II subgroup than in the NYHA III/IV subgroup, although the incremental gain in QALYs in the SCAD group compared to the standard care group was greater in the NYHA III/IV group. Costs were somewhat higher in the NYHA III/IV group and the cost savings in the SCAD group compared to the standard care group lower in this group. The ICER was higher in the NYHA I/II group (€7500) than in the NYHA III/IV group (€5176).

The outputs of the scenario analyses in which the parameters defining the structure of the model were varied are presented in Table 7. When a 5 years time horizon was used, the SCAD setting was dominant over standard care, being both less expensive and more effective. When the horizon was extended over the entire patient’s lifetime, both costs and utilities were higher than in the base case analysis, and the ICER increased by around 20% from €6491/QALY to €8151/QALY.

**Table 7:** Scenario analyses varying the model parameters

	Life years	QALYs	Total costs (€)	ICER (€/LY)	ICER (€/QALY)
*Base case*					
SCAD	6.03	4.41	35,177		
SC	5.11	3.75	30,932	4579	6491
Difference	0.93	0.65	4245		
*Five-year time-horizon*					
SCAD	4.03	2.92	21,290		
SC	3.58	2.61	21,372	SCAD	Dominant
Difference	0.45	0.31	-82		
*Lifetime time-horizon*					
SCAD	7.49	5.51	45,451		
SC	6.22	4.60	38,055	5841	8151
Difference	1.27	0.91	7396		
*All patients previously hospitalised at model entry*					
SCAD	5.79	4.23	36,527		
SC	4.73	3.47	32,679	3609	5082
Difference	1.07	0.76	3848		
*SCAD participation for 10 years*					
SCAD	6.29	4.59	39,023		
SC	5.11	3.75	30,932	6829	9680
Difference	1.18	0.84	8095		

*ICER* incremental cost-effectiveness ratio; *LY* life-years; *QALY* quality-adjusted life year; *SC* standard care; *SCAD* *Suivi Clinique A Domicile*

In the analysis in which all patients entered the model in the ‘not hospitalised after one previous hospitalisation’ state (Table 7), the ICER was around 20% lower than for the base case (€5082/QALY). This difference was due both to a reduction in cost and to an increase in life-years gained. This scenario analysis is expected to reflect real-life practice more accurately than the base case analysis.

In the analysis in which patients participated in SCAD for the entire 10 years lifetime of the model (Table 7), the additional cost of the SCAD setting over standard care was twice as high as in the base case, but the number of QALYs gained was also somewhat higher. In consequence, the ICER in this scenario analysis was also higher than in the base case (€9680/QALY).

## Discussion

In this cost-effectiveness evaluation of SCAD, a home-based interactive telemonitoring service for HF in France, we identified significant clinical benefits (one life-year gained in the ten years following initiation of the programme in the base case of the model) for a relatively modest cost (€4245 over the lifetime of the model). The estimated ICER was €6491/QALY. An intervention is considered to be cost-effective when the estimated ICER is below the willingness-to-pay threshold in the given country. While there is no formal willingness-to-pay threshold in France, a threshold of €150,000/QALY has been proposed [32]. The ICER for the SCAD programme is well below this threshold and, in the probabilistic sensitivity analysis, would be below a willingness-to-pay threshold of €11,800 in 90% of simulations.

In order to identify in which type of patient monitoring in the SCAD programme might be more effective, we performed two scenario analyses in different subgroups of patients. We observed modest differences in the estimated ICER of < 15% between the three LVEF subgroups. These differences can be explained by the fact that, in our cohort, these subgroups present different risk profiles. The mrEF group, which has the highest ICER, has the most favourable risk profile, being younger, more frequently less severe (NYHA class I/II), and less frequently previously hospitalised for HF, compared to the other two groups (see Additional file 1). For this reason, these patients are less likely to be hospitalised or die, and the absolute incremental benefit of SCAD participation compared to standard care will be numerically lower. This translates into a higher ICER in this group. In contrast, patients with preserved ejection fraction are on average older and have had more previous hospitalizations compared to patients with reduced ejection fraction. The ICER is correspondingly lower in this subgroup. Nonetheless, the observed differences in ICER between the three LVEF groups are small compared to the standard deviation of the ICER for the base case determined in the probabilistic sensitivity analysis (€3883). However, it should be noted that, in the absence of data on ejection fraction in the SNDS, from which transition probabilities were derived, the same transition probabilities were applied for all three ejection groups. If these probabilities in fact differ between groups, then this would have an impact on the estimated ICERs in these patient subgroups.

Differences in the cost-effectiveness of the SCAD programme between subgroups of different severity were more substantial, €7500 in patients with NYHA class I/II and €5176 in those with NYHA class III/IV HF. Again, this difference is principally driven by the risk of hospitalisation and death, and suggests that the SCAD programme is most cost-effective in patients with more severe heart disease. This is consistent with findings from the OSICAT study, a randomised clinical trial in France comparing telemonitoring with the Chronic Care Connect programme [33], which also reported that the largest clinical benefits of telemonitoring are observed in patients with more severe HF.

The ICER for the SCAD programme increases as the lifetime of the model is extended. This relationship can probably be explained by longer survival of patients included in the programme. These survivors continue to consume healthcare resources for longer, increasing total cost, whereas in standard care, more rapid attrition limits the cost of healthcare over the long term. However, even if the time horizon of the model is extended to the patient’s entire life expectancy, the ICER remains well below €10,000/QALY. When the time-horizon of the model was limited to five years, which is the longest period for which actual data on the impact of the SCAD programme on benefits and costs are available [19], the SCAD strategy is actually dominant over the standard care strategy, being both more effective in terms of QALYs gained and cost-saving. It is difficult to estimate accurately the long-term cost-effectiveness of the SCAD programme due to uncertainty over how long the clinical benefits in terms of reduced hospitalisation and mortality may be expected to last. In the model, a conservative approach was taken in which the benefits of the SCAD programme were limited to the five-year period for which data were available. It is possible that as more long-term data becomes available, estimations of the ICER for time horizons beyond five years may change.

The fourth scenario analysis in which all patients entered the model in the ‘not hospitalised after one previous hospitalisation’ state corresponds to how the SCAD programme currently operates, where only patients who are hospitalised are offered participation in the programme. The base case represented a conservative hypothesis, where certain patients are never hospitalised are also included in the model, and who will contribute equally to both the standard care and the SCAD strategy. In the base case, this will have the effect of diluting incremental utility and cost differences between the two strategies. The lower ICER determined in the fourth scenario analysis may for this reason reflect more accurately the cost-effectiveness of the SCAD programme as it is operated today.

Although telemonitoring programmes such as SCAD bear a cost, which is attributable both to the cost of the programme itself and to higher management costs related to higher outpatient costs and longer survival of the patients, this cost is relatively small compared to the total cost of management of HF. In France, there are around 540,000 patients managed for chronic HF, who generated a total cost to national health insurance in 2013 of €1186 million [34]. Since the SCAD programme is dominant in the short term (between 1 and 5 years), making it available to all patients in France at the current cost (€470 for 6 months) would generate short-term savings from the reduction in hospitalizations. It should however be noted that implementation of the SCAD programme bears a specific cost relating to funding for the full-time involvement of a trained nurse dedicated to patient monitoring, patient coaching and alert management.

Comparison of the present findings with those of cost-effectiveness analyses performed in other countries is not straightforward, due to differences in the nature of the telemonitoring programme considered, and in how they are financed. Nonetheless, recent studies of intense telemonitoring programmes for HF have consistently shown them to be cost-effective. For example, a Markov model similar to the present one has been used to evaluate cost-effectiveness of telehealth programs for congestive heart failure in the context of the United States health system [14]. Inputs were derived from a meta-analysis of multiple home telemonitoring programmes [35]. At the five-year time horizon, the authors found that enrolment in such a programme would result in cost savings of $4456 with a gain of 0.50 life years. A later analysis in the US health system reported found that telemonitoring was most cost-effective in patients with severe HF (NYHA class III/IV) [36]. In the European context, data from the Trans-European Network–Home-Care Management System (TEN–HMS) study [37] were used in a Markov model involving transitions between different NYHA severity classes [11]. At the twenty-year time horizon, the ICER was estimated to be €12,479/QALY. Most recently, Vesterggard et al. have reported on the TeleCare North HF study in Denmark [16]. This is a telemedicine programme implemented by nurses with a therapeutic education component and remote monitoring of clinical data provided by the patient. This cost-effectiveness analysis was not a modelling study but used real data on utilities and costs collected from patients participating in the programme. This analysis found the telemonitoring strategy to be dominant over standard care, with a net monetary benefit of £5164 (approximately €6100) at a time-horizon of one year. Taken together, the findings of these different studies provide a strong argument that intensive telemedicine programmes are a cost-effective way to manage patients with HF in different healthcare systems, consistent with the findings of the present study. Remote monitoring of patients with HF can thus make a beneficial contribution to a value-based approach to funding health services [38].

The study presents certain limitations. The model inputs come from multiple published sources, principally the ODIN study [25] and the SCAD-SNDS cohort [19], and the different source populations may not be fully comparable. In addition, in the SCAD-SNDS cohort, which was used as the source of the relative risks of hospitalisation and mortality, there was no control group without home telemonitoring, and the event rates in the low adherence group were used to represent standard care. However, in the SCAD study, even low-level users had lower rehospitalisation rates compared to the period before joining the SCAD programme, suggesting that the low adherence group may gain some benefit from the programme compared to non-participants. For this reason, the incremental gain in QALYs compared to standard care may have been underestimated.

## Conclusions

Enrolment of patients into the SCAD programme is highly cost-effective. Extension of the programme to other hospitals and more patients would have a limited budget impact but provide important clinical benefits. This finding should also be taken into account in new public health policies aimed at encouraging a shift from inpatient to ambulatory care.

## Supplementary Information

**Additional file1**. Description of patients from the SCAD cohort

**Additional file2**. Kaplan–Meier survival curves stratified by level of SCAD use

**Additional file3**. Costs for healthcare resource consumption determined in the SCAD–SNDS linkage study.

**Additional file4**. Input data for the scenario analyses: clinical events

**Additional file5**. Relative risks

## Data Availability

All data and software used to construct this model are identified either in the manuscript or in cited source references.

## Abbreviations

CI: Confidence interval
DSA: Deterministic sensitivity analysis
EF: Ejection fraction
HF: Heart failure
HF-mrEF: Heart failure with mid-range ejection fraction
HF-pEF: Heart failure with preserved ejection fraction
HF-rEF: Heart failure with reduced ejection fraction
HR: Hazard ratio
ICER: Incremental cost-effectiveness ratio
LL: Lower limit
LVEF: Left ventricular ejection fraction
LY: Life year
mrEF: Mid range ejection fraction
NHI: National Health Insurance
NYHA: New York Heart Association class
PSA: Probabilistic sensitivity analysis
pEF: Preserved ejection fraction
QALYs: Quality-adjusted life years
QoL: Quality of life
rEF: Reduced ejection fraction
SC: Standard care
SCAD: Suivi Clinique A Domicile
SHIFT: Systolic HF treatment with the if inhibitor ivabradine trial
TEN–HMS: Trans-European Network–Home-Care Management System
TP: Transition probability
UL: Upper limit
