# Supplementary material for: A telemonitoring programme in patients with heart failure in France: a cost-utility analysis
Source: BMC Cardiovasc Disord. 2022 Oct 10;22:441. doi: 10.1186/s12872-022-02878-1 (PMC9549824; doi:10.1186/s12872-022-02878-1)
Supplement: Supplementary file 1 — Additional file1. Description of patients from the SCAD cohort [file 12872_2022_2878_MOESM1_ESM.docx]

A Telemonitoring Programme in Patients with Heart Failure in France: A Cost-Utility Analysis

Additional Material

**Additional Table 1** Description of patients from the SCAD cohort

***A. By age and NYHA stage***

|  | **Patient subgroup** | | | | **TOTAL** |
| --- | --- | --- | --- | --- | --- |
|  | < 70 years | | ≥ 70 years | |  |
|  | NYHA I/II | NYHA III/IV | NYHA I/II | NYHA III/IV |  |
| Number of patients | 216 | 101 | 154 | 114 | 585 |
| % of patients | 37% | 19% | 24% | 20% | 100% |
| **Demographics** |  |  |  |  |  |
| Mean age at enrolment (years) | 55.7 | 59.9 | 77.8 | 78.2 | 66. 3 |
| Gender (% men) | 75% | 80% | 73% | 72% | 75% |
| **Number of hospitalisations associated with heart failure in the previous year** | | | | | |
| None | 71% | 50% | 49% | 24% | 52% |
| One | 24% | 37% | 38% | 54% | 36% |
| Two or more | 5% | 13% | 13% | 22% | 12% |
| **Comorbidities** | | | | | |
| Diabetes | 22% | 26% | 30% | 42% | 29% |
| Chronic kidney disease | 9% | 20% | 19% | 24% | 16% |
| **User type** | | | | | |
| Low user | 0% | 0% | 0% | 0% | 0% |
| Intermediate user | 55% | 56% | 50% | 38% | 50% |
| High user | 45% | 44% | 50% | 63% | 50% |
| **Left ventricular ejection fraction** | | | | | |
| Preserved ejection fraction | 19.0% | 17.8% | 27.3% | 27.2% | 22.6% |
| Mid-range ejection fraction | 28.2% | 26.7% | 26.6% | 21.1% | 26.2% |
| Reduced ejection fraction | 52.8% | 55.5% | 46.1% | 51.8% | 51.3% |

***B. By ejection fraction***

| **Variable** | **Category** | **Value** | | |
| --- | --- | --- | --- | --- |
| ***Population*** |  | ***pEF*** | ***mrEF*** | ***rEF*** |
| Age group | <70 years | 48.1% | 60.6% | 53.6% |
|  | ≥70 years | 51.9% | 39.4% | 46.4% |
| Severity | NHYA I/II | 62.0% | 65.3% | 55.0% |
|  | NHYA III/IV | 38.0% | 34.67% | 45.0% |
| Comorbidities | Diabetes mellitus | 37.4% | 31.8% | 23.5% |
|  | Chronic kidney disease | 14.6% | 16.6% | 17.6% |
| Extent of use of programme | Intermediate | 48.1% | 45.1% | 53.2% |
|  | High | 51.9% | 54.9% | 46.8% |
| Number of hospitalisations for HF | 0 in previous 12 mo | 40.8% | 56.6% | 53.2% |
|  | 1 in previous 12 mo | 46.0% | 32.1% | 34.3% |
|  | ≥2 in previous 12 mo | 13.2% | 11.4% | 12.5% |
