# Supplementary material for: A telemonitoring programme in patients with heart failure in France: a cost-utility analysis
Source: BMC Cardiovasc Disord. 2022 Oct 10;22:441. doi: 10.1186/s12872-022-02878-1 (PMC9549824; doi:10.1186/s12872-022-02878-1)
Supplement: Supplementary file 2 — Additional file2. Kaplan–Meier survival curves stratified by level of SCAD use [file 12872_2022_2878_MOESM2_ESM.docx]

A Telemonitoring Programme in Patients with Heart Failure in France: A Cost-Utility Analysis

Additional Material

**Additional File 2** Kaplan-Meier survival curves stratified by level of SCAD use

**A. Hospitalisation for heart failure**

**B. Death**


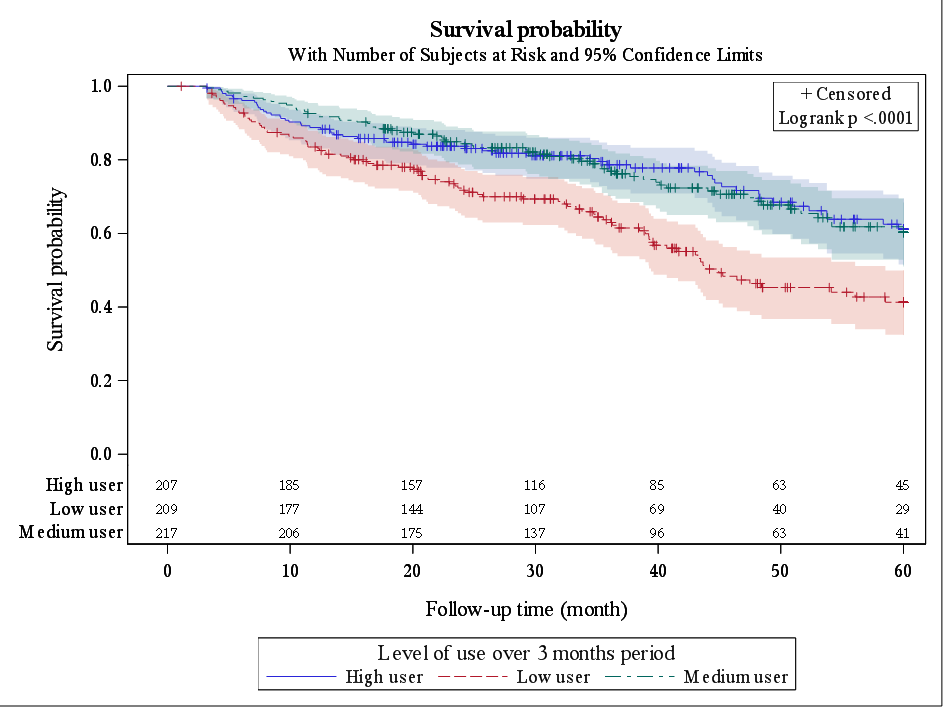


Red curve: low use; turquoise curve: intermediate use; blue curve: high use.
