# Supplementary material for: A telemonitoring programme in patients with heart failure in France: a cost-utility analysis
Source: BMC Cardiovasc Disord. 2022 Oct 10;22:441. doi: 10.1186/s12872-022-02878-1 (PMC9549824; doi:10.1186/s12872-022-02878-1)
Supplement: Supplementary file 3 — Additional file3. Costs for healthcare resource consumption determined in the SCAD–SNDS linkage study. [file 12872_2022_2878_MOESM3_ESM.docx]

A Telemonitoring Programme in Patients with Heart Failure in France: A Cost-Utility Analysis

Additional Material

**Additional Table 3** Costs for healthcare resource consumption determined in the SCAD – SNDS linkage study.

|  | **Management of heart failure: Per capita costs (€)** | |  |
| --- | --- | --- | --- |
|  | **12 months before** | **12 months after** | **p-value** |
|  | **N = 528** | **N = 528** |  |
| **COMMUNITY CARE (aggregated costs)** |  |  | <0.0001 (i) |
| Mean (standard deviation) | 6,026.3 (6,790.2) | 7,095.3 (7,376.3) |  |
| Median / Min / Max | 4,177.7 - 0.0 – 81,178.5 | 4 671.5 - 0.0 – 70,456.8 |  |
| **Physician consultations** |  |  | 0.0005 (i) |
| Mean (standard deviation) | 1,188.0 (1,472.8) | 1,318.5 (1,923.8) |  |
| Median / Min / Max | 829.9 - 0.0 – 14,743.4 | 966.8 - 0.0 – 25,136.1 |  |
| **Nursing and other paraclinical care** |  |  | <0.0001 (i) |
| Mean (standard deviation) | 363.0 (1,120.3) | 538.8 (1,632.1) |  |
| Median / Min / Max | 68.4 - 0.0 – 12,501.4 | 114.3 - 0.0 – 24,573.6 |  |
| **Pharmaceuticals and blood products** |  |  | <0.0001 (i) |
| Mean (standard deviation) | 1,962.9 (5,062.1) | 2,380.5 (4,704.8) |  |
| Median / Min / Max | 1,099.4 - 0.0 – 78,019.2 | 1,384.8 - 0.0 – 48,944.6 |  |
| **Medical devices** |  |  | <0.0001 (i) |
| Mean (standard deviation) | 604.8 (1,192.3) | 767.9 (1,339.5) |  |
| Median / Min / Max | 141.8 - 0.0 – 10,514.8 | 240.0 - 0.0 – 10,079.3 |  |
| **Transportation** |  |  | 0.8230 (i) |
| Mean (standard deviation) | 945.0 (2,108.1) | 1,215.7 (2,556.5) |  |
| Median / Min / Max | 229.3 - 0.0 – 21,881.2 | 195.3 - 0.0 – 17,423.6 |  |
| **Dental care** |  |  | 0.0945 (i) |
| Mean (standard deviation) | 93.6 (417.6) | 149.8 (529.0) |  |
| Median / Min / Max | 0.0 - 0.0 – 6,792.1 | 0.0 - 0.0 – 5,713.8 |  |
| **Laboratory tests** |  |  | <0.0001 (i) |
| Mean (standard deviation) | 412.6 (410.9) | 517.0 (407.9) |  |
| Median / Min / Max | 310.0 - 0.0 – 3,844.8 | 423.9 - 0.0 – 3,170.1 |  |
| **Other services** |  |  | 0.0002 (i) |
| Mean (standard deviation) | 456.3 (1,738.8) | 207.0 (1,202.4) |  |
| Median / Min / Max | 0.0 - 0.0 – 21,004.1 | 0.0 - 0.0 – 14,046.5 |  |

1. Wilcoxon test for matched analysis, (k) McNemar CHI-square test

| **Average cost of hospitalisation per capita (€)** | | | |
| --- | --- | --- | --- |
| **All users** | **Low users** | **Intermediate users** | **High users** |
| 6,540.6 | 7,366.0 | 6,065.2 | 5,783.1 |

| **Average cost of palliative care (€)** | | |
| --- | --- | --- |
| **3^rd^ month before death** | **2^nd^ month before death** | **Month before death** |
| 1,872 | 10,981 | 8,659 |
