# Supplementary material for: A telemonitoring programme in patients with heart failure in France: a cost-utility analysis
Source: BMC Cardiovasc Disord. 2022 Oct 10;22:441. doi: 10.1186/s12872-022-02878-1 (PMC9549824; doi:10.1186/s12872-022-02878-1)
Supplement: Supplementary file 4 — Additional file4. Input data for the scenario analyses: clinical events [file 12872_2022_2878_MOESM4_ESM.docx]

A Telemonitoring Programme in Patients with Heart Failure in France: A Cost-Utility Analysis

Additional Material

**Additional Table 4** Input data for the scenario analyses: clinical events

|  | Base case analysis | pEF subgroup | mrEF subgroup | rEF subgroup | Source |  |
| --- | --- | --- | --- | --- | --- | --- |
| Transition probabilities for hospitalization (monthly) | | | | | | |
| 0 previous hospitalization to 1 | 0.006465718 | 0.00579124 | 0.003756865 | 0.007957855 | ODIN |  |
| 1 previous hospitalization to 2 | 0.016537578 | 0.016537578 | 0.016537578 | 0.016537578 | Feldman et al. |  |
| 2 previous hospitalizations to 3+ | 0.016537578 | 0.016537578 | 0.016537578 | 0.016537578 | Feldman et al. |  |
| Transition probabilities for mortality | | | | | | |
| 0 previous hospitalization to death | 0.00644676 | 0.006530267 | 0.005963319 | 0.006632359 | ODIN |  |
| 1 previous hospitalization to death | 0.008465564 | 0.008844895 | 0.008307781 | 0.008382805 | ODIN |  |
| 2 previous hospitalizations to death | 0.018058486 | 0.016834791 | 0.017131718 | 0.018972190 | ODIN |  |
| 1^st^ hospitalization to death | 0.008465564 | 0.008844895 | 0.008307781 | 0.008382805 | Assumption: same as 1 previous hospitalization to death |  |
| 2^nd^ hospitalization to death | 0.018058486 | 0.016834791 | 0.017131718 | 0.018972190 | Assumption: same as 2 previous hospitalizations to death |  |
|  | Base case analysis | pEF subgroup | mrEF subgroup | rEF subgroup | Source |  |
| Transition probabilities’ adjustments for hospitalisation (RR) | | | | | | |
| < 70 years & NYHA I/II | 0.79 | 0.77 | 0.78 | 0.81 | ODIN |  |
| < 70 years & NYHA III/IV | 1.43 | 1.60 | 1.52 | 1.32 | ODIN |  |
| ≥ 70 years & NYHA I/II | 0.74 | 0.69 | 0.73 | 0.78 | ODIN |  |
| ≥ 70 years & NYHA III/IV | 1.32 | 1.36 | 1.42 | 1.27 | ODIN |  |
| Transition probabilities for mortality | | | | | | |
| < 70 years & NYHA I/II | 0.76 | 0.74 | 0.88 | 0.71 | ODIN |  |
| < 70 years & NYHA III/IV | 1.48 | 1.68 | 1.28 | 1.49 | ODIN |  |
| ≥ 70 years & NYHA I/II | 0.71 | 0.66 | 0.85 | 0.66 | ODIN |  |
| ≥ 70 years & NYHA III/IV | 1.36 | 1.39 | 1.23 | 1.40 | ODIN |  |
