# Supplementary material for: A telemonitoring programme in patients with heart failure in France: a cost-utility analysis
Source: BMC Cardiovasc Disord. 2022 Oct 10;22:441. doi: 10.1186/s12872-022-02878-1 (PMC9549824; doi:10.1186/s12872-022-02878-1)
Supplement: Supplementary file 5 — Additional file5. Relative risks [file 12872_2022_2878_MOESM5_ESM.docx]

A Telemonitoring Programme in Patients with Heart Failure in France: A Cost-Utility Analysis

Additional Material

**Additional Table 5** Relative risks

| **Relative risk: high-intermediate vs low users** | HR | 95%CI-LL | 95%CI-UL |  |
| --- | --- | --- | --- | --- |
| **Death** | | | | |
| All type of ejection fraction | 0.535 | 0.407 | 0.704 |  |
| <40% - heart failure with reduced ejection fraction (HF-pEF) | 0.522 | 0.348 | 0.784 |  |
| 40-49% - heart failure with mid-range ejection fraction (HF-mrEF) | 0.546 | 0.312 | 0.956 |  |
| >=50% - heart failure with preserved ejection fraction (HF-pEF) | 0.490 | 0.289 | 0.832 |  |
| **Hospitalisation for Heart Failure** | | | | |
| All type of ejection fraction | 0.500 | 0.373 | 0.670 |  |
| <40% - heart failure with reduced ejection fraction (HF-rEF) | 0.540 | 0.356 | 0.819 |  |
| 40-49% - heart failure with mid-range ejection fraction (HF-mrEF) | 0.392 | 0.206 | 0.745 |  |
| >=50% - heart failure with preserved ejection fraction (HF-pEF) | 0.415 | 0.231 | 0.748 |  |

CI: confidence interval: EF: ejection fraction; HR: hazard ratio; LL: lower limit; UL: upper limit.
